# Nanomaterials for the Diagnosis and Treatment of Inflammatory Arthritis

**DOI:** 10.3390/ijms22063092

**Published:** 2021-03-18

**Authors:** Seyedeh Maryam Hosseinikhah, Mahmood Barani, Abbas Rahdar, Henning Madry, Rabia Arshad, Vahideh Mohammadzadeh, Magali Cucchiarini

**Affiliations:** 1 Nanotechnology Research Center, Pharmaceutical Technology Institute, Mashhad University of Medical Sciences, Mashhad 91886-17871, Iran; mhosseinikhah@yahoo.com; 2 Department of Chemistry, Shahid Bahonar University of Kerman, Kerman 761691411, Iran; mahmoodbarani7@gmail.com; 3 Department of Physics, Faculty of Science, University of Zabol, Zabol 538-9861, Iran; 4 Center of Experimental Orthopaedics, Saarland University Medical Center, D-66421 Homburg/Saar, Germany; henning.madry@uks.eu; 5 Department of Pharmacy, Quaid-i-Azam University, Islamabad 45320, Pakistan; rabia.arshad@bs.qau.edu.pk; 6 Department of Pharmaceutical Nanotechnology, School of Pharmacy, Mashhad University of Medical Science, Mashhad 91886-17871, Iran; mohammadzadehv971@mums.ac.ir

**Keywords:** nanodiagnosis, nanotreatments, inflammatory arthritis

## Abstract

Nanomaterials have received increasing attention due to their unique chemical and physical properties for the treatment of rheumatoid arthritis (RA), the most common complex multifactorial joint-associated autoimmune inflammatory disorder. RA is characterized by an inflammation of the synovium with increased production of proinflammatory cytokines (IL-1, IL-6, IL-8, and IL-10) and by the destruction of the articular cartilage and bone, and it is associated with the development of cardiovascular disorders such as heart attack and stroke. While a number of imaging tools allow for the monitoring and diagnosis of inflammatory arthritis, and despite ongoing work to enhance their sensitivity and precision, the proper assessment of RA remains difficult particularly in the early stages of the disease. Our goal here is to describe the benefits of applying various nanomaterials as next-generation RA imaging and detection tools using contrast agents and nanosensors and as improved drug delivery systems for the effective treatment of the disease.

## 1. Introduction

Inflammatory arthritis is an autoimmune heterogenous group of joint disorders associated with pain, stiffness, bone erosion, synovial hyperplasia, and progressive joint destruction [1,2] that aggravates prevailing inflammation to the associated connective tissues, lungs, heart, and skin [3]. The most alarming situation in inflammatory arthritis is the irreversible inflammatory damage [4,5]. Inflammation in arthritis is linked to either any infection or any foreign organism like bacteria, virus, and fungi [6]. Inflammation is the prime protective feature of the immune system in preventing from underlying pathogenic infections or injury [7]. Normal inflammatory processes of host immune cells in maintaining homeostasis accounts for the mucosal, connective, and epithelial barriers and proinflammatory signaling [8]. All these three natural mechanistic processes help in limiting the overburden of notorious pathogenic bacteria and overactivated immune response [9]. However, any physiological change in the body due to the various etiological factors like stress, genetics, and age leads to an imbalance in homeostasis and the penetration of pathogenic bacteria in the connective tissues and synovial joints, contributing to exaggerated inflammation and pathogenesis of the disease [10]. Other reasons for inflammatory arthritis may include a reduction in the amount of cartilage, the tissue that withstands shock and stress in the joint [10]. Moreover, inflammatory arthritis can be described in various types, i.e., ankylosing spondylitis where inflammation occurs via fusion of small bones of the spinal vertebrae, making spine hard and less flexible [11]. In some cases, ribs can also be affected and can cause difficult breathing [12]. Similarly, gout can be developed due to increased levels of uric acid and defined as inflammation in the joints of feet and toes, resulting in intense pain and a burning sensation [13]. Moreover, Lyme disease is characterized by the swelling of joints caused by infectious bacterial agents [14]. RA is the most common type of chronic inflammatory arthritis that can affect joints and damages variety of body systems and blood vessels [15,16]. The prevention of inflammatory arthritis is more effective compared with its treatment as lifestyle modifications, weight loss, and antioxidant rich food are highly effective in mitigating the onset of inflammatory arthritis [17]. As far as the treatment of inflammatory arthritis is concerned, emphasis is placed on the management of pain using heating and ice packs for soothing [18]. However, other practical sorts of treatment for inflammatory arthritis include medications like analgesics and non-steroidal anti-inflammatory drugs (NSAIDs) such as aspirin, celecoxib, diflunisal, ibuprofen, and naproxen sodium [19]. Menthol-based analgesic creams and immunosuppressants are also common modalities used against inflammatory arthritis [20,21]. NSAIDs function by restricting prostaglandin synthesis by inhibiting the enzyme cyclooxygenase (COX), thus reducing swelling and pain [22]. In severe conditions where medications remain ineffective, surgery can be performed to replace the joint with an artificial one, especially for hips and knees [23]. Physical therapy for the treatment of inflammatory arthritis is also common in some group of patients and involves muscle strengthening around the affected joint [24]. However, administration of medications through the oral and parenteral routes is limited due to low bioavailability, rapid metabolism, poor absorption, first-pass effect, and serious adverse effects [25]. Several diagnosis techniques for inflammatory arthritis includes blood tests like erythrocyte sedimentation rate (ESR) to assess the levels of inflammation in the body, C-reactive protein (CRP), and complete blood count (CBC), as well as joint scans and X-rays [15]. The diagnosis of inflammatory arthritis is a challenging issue due to high time gaps between the incidence of the disease and the possibility of detecting specific markers [26].

Nanomedicine has revolutionized the field of medicine and significantly proved to ameliorate the pharmacological and pharmacokinetic patterns of the conventional medications in the treatment of inflammatory arthritis [27,28,29]. Nanomaterials are sophisticated and smart systems to enhance drug delivery [30]. Therefore, nanoparticles (NPs) are attractive compounds to stabilize and encapsulate anti-inflammatory drugs, increasing their solubility, half-life, therapeutic index, safety, and efficacy [31,32]. At a higher level, NPs derived from smart formulations may also prompt targetability potential with specific receptors [33,34]. Several nanoformulations enriched with enhanced drug delivery in treating inflammatory arthritis include NSAID-based metallic and polymeric NP conjugates (chitosan-dexamethasone NPs, i.e., CS-Dex NPs), (hyaluronic-gold-tocilizumab NPs), human serum albumin NPs (arginine-glycine aspartic acid attached with gold nano half-shells conjugated with methotrexate (MTX)), solid lipid NPs (curcumin CUR-loaded SLNs), surface-engineered liposomes, nanoemulsions (quercetin-loaded nanoemulsion-based gel and intra-articular MTX-associated nanoemulsion) and nanomicelles, and herbal CUR-based NPs [35]. These NPs can be more effective in the size range of 100 to 400 nm. NPs can also be decorated with certain antibodies to specifically target the macrophages, playing a major role in inflammatory processes in arthritis [36,37,38]. Nanomedicine has also been largely involved in the detection of inflammatory arthritis with higher sensitivity, cost-effectiveness, and minimized overdiagnosis owing to its high specificity. Nanomaterial drug delivery is advantageous compared with conventional therapy because of its enhanced targeting specificity via controlled drug release, its increased ability to solubilize hydrophobic drugs, its synergistic combinatorial chemistry, and superior ability for drug delivery [39,40]. Advanced in vivo molecular imaging approaches like magnetic resonance imaging (MRI) can take images of the internal joint and cartilage [41,42]. Contrast agents used in MRI help in the differentiation between normal as well as abnormal tissues via magnetic signals [42]. Moreover, superparamagnetic iron oxide nanoparticles (SPIONs), gold, and CS-glyco-NPs (SPIONs) are captured by monocytes as well as macrophages and are transported to the inflamed tissues. These NPs allow for very high resolution, sensitive detection of discrete forms of inflamed joints and quantification via treating with Dex [43]. SPIONs have the remarkable ability to exhibit favorable non-toxicological profiles [42].

With this in mind, as a continuous effort related to the synthesis of nanomaterials and investigation of their potential in biological applications [44,45,46,47,48,49,50,51,52,53,54,55,56,57,58], we currently review here different nanomaterials applied to diagnosis and treatment of inflammatory arthritis.

## 2. Nanomaterials for the Diagnosis of Inflammatory Arthritis

### 2.1. Traditional Approaches

The disease is difficult to diagnose as there are more than 100 various kinds of arthritis and among the various disorders affecting the joints, several symptoms are identical [59]. The following classes will usually be defined as arthritis: inflammatory arthritis, degenerative arthritis, infectious arthritis, and metabolic arthritis. [60]. The most common form of arthritis is the inflammatory arthritis [61]. Historically, for the diagnosis of inflammatory arthritis, some common laboratory assays and imaging methods have been carried out. Popular laboratory tests include the detection of blood antinuclear antibodies, arthrocentesis (collection and testing of synovial fluid), complement tests, and blood cell counts (white and red blood cells, and platelets) [62,63]. For example, the sedimentation rate of erythrocytes or ESR determines how easily red blood cells deposit at the bottom of a test tube. When there is inflammation in the body, the amount of ESR increases [64]. The amount of red blood cells present in a sample of blood is determined by hematocrit or packed cell volume (PCV). Low red blood cell levels (anemia) are frequent in patients with arthritis [65]. The rheumatoid factor (RF) monitors the presence of an antibody in most patients with RA [66]. Interestingly, for inflammatory arthritis, uric acid and CRP increase in gout [67]. On the other hand, biomarkers may allow for the diagnosis of inflammatory arthritis at early stages of the disease. The ACR/EULAR 2010 criteria for the diagnosis of RA focus on the detection of RF and of antibodies against cyclic citrullinated proteins (anti-CCP), while early diagnosis may also include antibodies against carbamylated proteins (anti-CarP), mutated citrullinated vimentin antibodies (anti-MCV), cartilage oligomeric matrix protein (anti-COMP), serum calprotectin, and 14-3-3 eta protein.

Imaging methods, on the other hand, may provide a better understanding of the processes that occur in the joint(s) during inflammatory arthritis. X-rays, ultrasound (US), MRI, and arthroscopy are imaging techniques that can be employed for inflammatory arthritis [68,69]. X-rays reveal changes in the joints and damage to the bone seen in inflammatory arthritis. In order to see the condition of synovial tissue, ligaments, tendons, and joints, US is based on sound waves without radiation. MRI photographs are even more accurate than X-rays, showing joint damage, including in the muscles and tendons [70]. Arthroscopy consists of a thin tube (arthroscope) carrying a flashlight and a camera to peek through the joint. It is used to diagnose any joint debilitating and/or arthritic alterations, to classify bone disorders and tumors, and to evidence the severity of bone inflammation and pain [71,72].

All methods have many drawbacks such as low precision, poor image resolution, and high cost, despite the extensive use of conventional methods in the diagnosis of arthritis. However, important progress in nanomedicine has been made recently and new platforms for high precision diagnosis of inflammatory arthritis can be introduced by nanotechnology [73,74].

### 2.2. Nanoimaging

**Figure 1:**
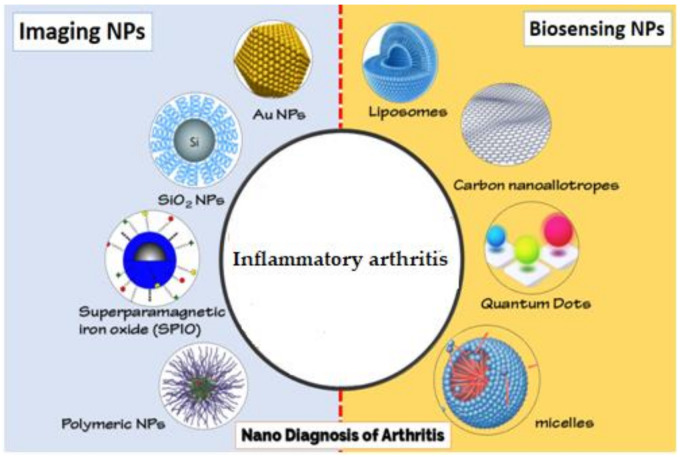
Different nanoparticles (NPs) for imaging and biosensing of inflammatory arthritis.

A selection of imaging methods contribute in the diagnosis and evaluation of inflammatory arthritis, although the proper assessment of arthritis, especially in the early stages of the disease, can be problematic [75]. Various studies are therefore ongoing to improve the sensitivity and accuracy of imaging methods to facilitate early stage diagnosis of inflammation arthritis [76]. In the biomedical area, the engineering of nanoscale materials is increasingly used to fulfill this goal [77]. In the following paragraphs, the use of SPIO, gold, polymeric NPs, and multimodal nanomaterials (cerium and silica NPs) will be discussed that could have promising biomedical applications in the imaging of inflammatory arthritis (Figure 1).

For both preclinical animal models and human clinical trials, SPIONs have been used as contrast agents in a wide variety of diseases such as coronary artery disease, malformation, muscular dystrophy, cancer, inflammation, transplant failure, and arthritis [78]. SPIONs are able to picture different types of infection at the cellular level like RA [79]. For example, Chih-Lung et al. [80] prepared targeted SPIONs for in vivo MRI of T cells in RA via attachment of a monoclonal anti-CD3 antibody to carboxylated-polyethylene glycol (PEG)-SPION (IOPC-CD3). Serial MRI analysis showed a selective reduction of the S/N ratio of IOPC-CD33-infused femoral growth plates in the collagen-induced arthritis (CIA) model of RA in rats, in agreement with immunohistochemical findings showing that the aggregation of T cells and SPIONs will be in the target area.

Gold nanostructures including injectable auranofin for RA have been used for medicinal purposes for decades [81]. Gold nanostructures have been widely evaluated in a number of imaging techniques, including CT, MRI, Raman spectroscopy, fluorescence, and photoacoustic imaging owing to its unique light scattering abilities and configurable surface plasmon resonance [82]. The desirable imaging features and simplicity of gold nanomaterial preparation make it a good substrate for selective inflammation and arthritis imaging [83]. Marc et al. [84] prepared enlarged gold nanorods with a sharp absorption at 1064 nm and modified them using infliximab and certolizumab antibodies to target tumor necrosis factor alpha (TNF-α). This nanoprobe was used to define inflammation through optoacoustic molecular imaging in arthritic mouse knees. Using a fast-scanning optoacoustic imaging device based on a pulsed Nd:YAG laser and a single centered US transducer, they demonstrated a longitudinal improvement of optoacoustic signal magnitudes after injection of infliximab- but not certolizumab-modified and PEGylated control particles on arthritic and healthy control mice.

In light of their good light trapping nature, bioactivity, and configurable adsorption capacities, the use of conjugated polymer nanomaterials solved the limitations of traditional contrast agents, being superior imaging substances [85]. For example, a new near-infrared (NIR)-II conjugated polymer nanoprobe (PNP) has been synthesized by Chen et al. [86] for use in a CIA animal model. Tocilizumab-loaded nanopolymers demonstrated high-resolution images of swollen and cartilage tissue using NIR-II PMI of the RA joint tissue, indicating its monitoring ability for noninvasively tracking the development of RA disease. In related work, Hieu et al. [87] employed a combination of NIR imaging and 19F MRI with labeled NP injection in vitro and in CIA mice. The NPs were made from poly-(lactic-co-glycolic acid) (PLGA)-PEG-folate (folate-NP) or from PEG-block-PLGA, loaded with indocyanine green (ICG) and perfluorooctyl bromide (PFOB). In the inflamed synovial membrane/fluid and the pannus of inflammatory vascular tissue in RA-affected joints, an excess of macrophages was identified. At the early point of time (2 h), the existence of folate as a targeting ligand greatly enhanced the NIR signal from inflamed tissue. In another study, Shuyi et al. [88] designed a cartilage-targeted cationic nanoprobe to enhance photoacoustic imaging (PAI) based on poly-l-lysine-melanin (PLL-M)-NPs to track the development of arthritis. The in vitro analysis displayed the capability of PLL-M-NPs to identify various high-sensitivity anionic glycosaminoglycan (GAG) concentrations. Responsive and consistent visualization of arthritis development was supported by the use of nanoprobe-enhanced PAI to recognize changes in GAG material.

Multimodal imaging modalities in which the strengths of more than one imaging technique are combined can also be used for NPs. For example, as a multimodal imaging agent, silica-based NPs are attractive because of their biocompatibility, photostability, multivalent binding capability, and biodistribution [89]. Nanoceria, cerium oxide-based NPs, are another form of biocompatible NPs applied in arthritis multimodal imaging [90].

### 2.3. Nanodiagnosis

The prevalence of arthritis since the start of the post-industrial period has alarmingly risen [91]. Historically, the traditional medical diagnosis of arthritis is focused on symptoms of pain and decreased function and computed tomography (joint disturbances) that frequently appear late in the course of the disease [92]. The analysis of biological parameters may be an enticing and realistic alternative. For example, RF and anti-CCP are used for inflammatory arthritis diagnosis based on EULAR 2010 guidelines [91]. The most promising alternative for the future diagnosis and treatment of inflammatory arthritis are actually NPs [73]. Nanomaterials such as quantum dots, carbon nanoallotropes, micelles, and liposomes will be discussed in following paragraphs (Figure 1).

RA is characterized by the development of auto-antibodies, synovial inflammation, and degradation of bones and RF auto-antibodies are the most recognized biomarkers for arthritis as described above. To address this problem, Veigas et al. [93] developed a cost-effective and simple approach to detect and quantitatively measure the RF marker. This colorimetric nanosensor was based on crosslinking of the Au nanoprobe, resulting in extensive accumulation in the vicinity of the pentameric IgM RF. Nanoconjugate accumulation causes a change of color from red to purple that can be easily detected by the unaided eye. A limit of detection (LOD) of 4.15 UA/mL IgM RF was obtained by the nanoplatform.

In biological applications, organic–inorganic hybrid nanomaterials have been investigated because of their structural and compositional variations in each section. Due to its interparticle plasmonic pairing for photonics-based biosensor applications, a mixture of these hybrid NPs with metallic nanomaterial would be desirable [94]. Hwang et al. [94] prepared silver/gold (Ag@Au) core–shell NPs)-poly(aniline) hybrid nanostructures (CBCPHNs) for the early diagnosis of RA. Nanohybrids were used in surface-enhanced Raman scattering (SERS)-based multiplexed detection of auto-antibodies (RF IgM and anti-CCP). The LOD for RF IgM and anti-CCP was 0.93 IU/mL and 0.68 IU/mL, respectively. In animal studies, the relationship between mycoplasma pneumonia (MP) and RA has been confirmed for decades, with an increased risk of RA in patients with mycoplasma pneumonia [95]. Jia et al. [96] prepared Raman-based immunoassay strip for the accurate detection of MP contagion in blood samples. In this immunoassay, Au@Ag NPs were loaded with two layers of Raman dye 5,5′-dithiobis-(2-nitrobenzoic acid) (DTNB) as SERS tags. The LOD was of 0.1 ng/mL for RF, i.e., 100-fold more accurate than the colorimetric assay.

Cardiovascular diseases (CVDs) and RA are generally associated with human immunodeficiency virus (HIV) infection, a global public health issue [97]. Islam et al. [98] designed a unique biosensor based on graphene-based field-effect transistors to detect HIV and its associated diseases (CVDs and RA). In this study, amine-functionalized graphene (afG) was integrated with antibodies (anti-CCP for RA, anti-cTn1 for CVD, and anti-p24 for HIV) to sense several biomarkers. Via carbodiimide activation, the antibodies were conjugated covalently to afG. The nanosensor was highly susceptible and displayed a linearity for the p24, cTn1, and CCP biomarkers. The LOD was of 10 fg/mL for CCP and cTn1 and of 100 fg/mL for p24.

## 3. Nanomaterials for the Treatment of Inflammatory Arthritis

RA is one of the most common autoimmune and inflammatory progressive disorders diagnosed with several principal symptoms such as synovial joint damage and cartilage and bone tissue dramatic malformation [99]. Approximately 1.5 million people worldwide suffer from RA. The prevalence of this inflammatory disorder in women is almost three times higher than in men [100]. This inflammation could also develop in other tissues like the heart, lungs, kidneys, and pleura. Although there are some methods for the treatment of RA, none are reliably effective [99]. There are presently several groups of anti-arthritis therapeutic agents use for RA therapy, such as NSAIDs and disease-modifying anti-rheumatic drugs (DMARDs) which includes biologics like an anti-interleukin 6 (anti-IL-6) receptor and anti-TNF-α antibodies [101]. Although considered strong and effective drugs, their long-term consumption may cause serious side effects. One of the main problems associated with the use of these drugs is that they are widely distributed throughout the body, except at the site of disease and inflamed joints. For this reason, the treatment of chronic arthritis is often associated with the destruction to several important organs like the liver, kidneys, and lungs. Therefore, it is necessary to effectively deliver such drugs to the inflamed RA sites in order to increase their efficacy in managing arthritis [101].

Nanomaterials can provide adapted tools to address this issue. Nanomaterials have been extensively employed to increase the bioavailability, bioactivity, pharmacokinetics, and pharmacodynamics of drugs against RA. It is therefore necessary to expand and investigate new and suitable therapeutic agents for the treatment of RA that exactly and correctly target abnormal joints without damaging other, healthy tissues. A variety of studies employed NPs for the therapy of RA, including liposomes, polymeric NPs, niosomes, metal NPs, quantum dots, SLNs, and polymeric micelles. The findings of these investigations revealed the performance of these systems due to their specific physicochemical properties such as biocompatibility, lack of toxicity, ability to promote sustained drug release, and selective drug delivery to damaged and inflamed tissues in animal RA models [99,102].

### 3.1. Liposomes

**Figure 2:**
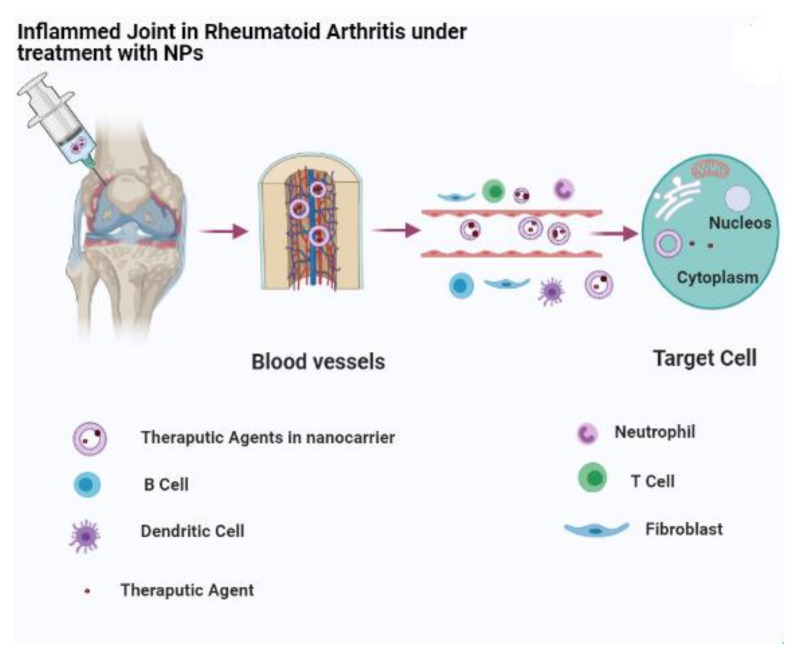
Liposomes as nanocarriers for the delivery of drugs in the treatment of RA.

Liposomes are considered successful nanovehicles formed by lipid bilayers surrounding an aqueous core for drug delivery strategy. They are well-known nanoscale systems with reduced toxicity and minor side effects for the administration of therapeutic agents. Liposomes as drug delivery nanocarriers have numerous benefits including a low immunogenicity and high biocompatibility, the ability of loading and conjugating both hydrophilic and lipophilic agents, and suitable sizes with physico-chemical features that can improve the stability and prolong the biological half-life of drugs while potentially delivering medicines specifically to damaged and inflamed joints (Figure 2) [103].

Although liposomes have certain advantages, there are two important challenges remain for a therapeutic application of conventional liposomes. First, the blood circulation time of liposomes is restricted as they can be rapidly removed by phagocytic cells in the liver. Second, liposomes typically do not have long-term stability under physiological conditions. The presence of these challenges may lead to the release of encapsulated drugs at off-target sites. Several factors may have effects on the instability of liposomes including the osmotic pressure, the hydrolysis of lipid, and a surfactant-induced decomposition. The polymerization process of lipids in bilayers has been evidenced as a powerful method to get the structural integrity of liposomes [5].

MTX is a drug commonly prescribed to treat RA. Due to the intrinsic adverse effects of this drug, loading of MTX in liposomes may be a suitable delivery method to diminish its toxicity while retaining its properties and efficacy. The encapsulation efficiency (EE) of MTX (or of any other type of drug) in liposomes is regulated by the features of the liposomes, including their aqueous volume and membrane rigidity. Encapsulation may also be affected by the hydrophilic/hydrophobic parts of a drug that may impact its capacity to interact with the liposome membrane bilayer. Loading of MTX in liposomes occurs via a passive procedure controlled by the ability of the liposome to trap the aqueous phase containing the drug. This procedure results in lower EE as drug retention is restricted to the size of the aqueous part in the liposome and to the solubility of the drug. In a recent work, Guimarães et al. [104] improved the loading of MTX in liposomes by reducing the initial formed solution (20% at a 1:1—*v/v*—organic:aqueous phase ratio) using a procedure based on ethanol injection, so that the aqueous volume outside the liposomes dissolved in ethanol was diminished, promoting the interaction between MTX and lipids to obtain a suitable size distribution and greater drug EE. They found a small size and suitable polydispersity index (PDI) with higher loaded MTX, the efficiency was more than 30% compared with conventional ethanol injection method. Results obtained by nuclear magnetic resonance (NMR) revealed bilateral connections between the drug and the main phospholipid through hydrogen bonding, increasing EE. The authors were thus capable of developing a new pre-concentration ethanol injection technique to achieve higher MTX encapsulation in liposomes, an important advance for the therapy of RA.

The presence of liposomes in the blood circulation for several hours is considered the main role in passive targeting, depending on several factors like hydrophobicity, surface charge, and particle size. The diameter of the liposomes is the basic factor that has an effect on blood circulation time and biological release after intravenous injection. Moreover, the diameter of liposomes influences their permeation via gaps in leaky synovial vessels and retention in damaged joints. Their targeting capability is basically ascribed to the leaky vasculature of the synovial fluid caused by proinflammatory cytokines such as IL-1β, IL-6, and TNF-α, resembling the boosted the effect of permeability and retention (EPR) in tumors. The size of the endothelial gap between cells is a specified range due to the adsorption of plasma proteins and the phagocytosis by the reticuloendothelial system (RES), but can differ between patients [103].

In recent study, Ren et al. [105] investigated the mechanisms of passive targeting of liposomes and indicated the effect of their biophysical and biochemical features on their retention time in the blood stream. The authors prepared liposomes with various sizes and surface charges and also used different PEG chain lengths (1, 2, and 5 kDa) and concentrations (5%, 10%, and 20% *w*/*w* of total fat by lipid film dispersion and extrusion) and assessed their targeting ability using an NIR fluorescence imaging arrangement. They next employed optimal liposome systems (charge, size, etc.) to deliver Dex in CIA rats. Pharmacodynamics studies revealed that Dex liposomes significantly increased the anti-arthritic effects of Dex in this RA model in vivo. In RA, when the wall of blood vessels becomes inflamed, the vessels may become weakened and enhance in size, or they become leaky in the inflamed joints. In passive targeting, the secretion of nanosize drug delivery carriers via the leaky vasculature and following inflammatory cell-mediated sequestration (ELVIS) can lead to their accumulation, especially in sites of inflamed joints, and to an increased anti-inflammatory efficacy [103]. In another study, Wang et al. [103] prepared polymerized stealth liposomes consisting of 1,2-bis (10,12-tricosadiynoyl)-sn-glycero-3-phosphocholine (DC_8,9_PC) and 1,2-distearoylsn-glycero-3-phosphoethanolamine PEG (DSPE-PEG 2000) using the thin film hydration procedure. To increase the integrity of the liposomes and enhance their blood circulation time, the authors used DC_8,9_PC molecules crosslinked in a bilayer of liposome by ultraviolet (UV) radiation and PEG chains in order to make a stealth layer, respectively. The biocompatible liposomes were then administered to arthritic rats, with effective mobilization in the damaged joints. Administration of Dex via encapsulation in such polymerized stealth liposomes suppressed the concentration of proinflammatory cytokines such as TNF-α and IL-1β in joint textures and reduced the swelling of inflamed and damaged joints, overall preventing further progression of RA. In addition, Shen et al. [106] prepared new thermosensitive liposomes based on dipalmitoyl phosphatidylcholine (DPPC), hydrogenated soyabean phosphatidylcholine (SPC), and cholesterol to load the aquatic-soluble drug sinomenine hydrochloride (SIN). The liposomal delivery systems with suitable particle size had great compatibility and storage stability, allowing to successfully prevent the release of SIN in the blood circulation before reaching target sites in RA rats upon full release via microwave hyperthermia. The thermosensitive liposome delivery systems enhanced the concentration of the drug at the inflamed site of RA by improved controlled release and reduced RA signs without side effects, especially when combining the SIN treatment with microwave hyperthermia as an optimized, combined therapy to possibly manage the clinical symptoms of RA.

### 3.2. Polymeric NPs

Polymeric NPs are being prepared from colloidal particles and the diameter ranges considered (1–1000 nm). In fact, polymeric NPs have a great potential in the medical field due to their advantageous properties such as biodegradability, biocompatibility, great synthetic flexibility, ability to be precisely tailored, and appropriate mechanical properties [107]. To prevent the macrophage uptake, the surface of NPs may be sheathed with stealth polymers like PEG, and as the PEG covering density and thickness enhance, the polymeric NP circulation time increases in the blood. Modification of NPs via PEGylation, a process of covalent conjugation that prevents removal from the reticuloendothelial system, or via conjugation with other small molecules (peptides, vitamins, and antibodies) can greatly prolong the circulation time of the systems in the blood and improve the efficacy of the anti-RA drug being delivered, such as NSAIDs, corticosteroids, DMARDs, small interfering RNAs (siRNAs), and therapeutic peptides [108]. Synthetic cationic polymers such as polyethylenemine (PEI), poly-L-lysine (PLL), and dendrimers are usually utilized to deliver nucleic acids such as DNA and interfering RNAs (RNAi) [109]. Among them, PEI is the most frequently employed because of numerous protonated amino functional groups, allowing for a higher cationic charge density at physiological pH that facilitates the attachment of nucleic acids via electrostatic adsorption [109].

Espinosa-Cano et al. [110] demonstrated the benefits of using polymeric NPs conjugated with naproxen and Dex to decrease inflammation and prevent IL-12 expression in macrophages. Note that IL-12 and IL-23 recently appeared as therapeutic targets in the therapy of long-lasting inflammatory disorders in which T cells are the primary dysfunctional immune cells, via either COX-dependent or COX-independent regulation mechanisms. The authors prepared anti-inflammatory polymeric NPs by mixing Dex and ketoprofen (Ket) with suitable chemical and physical properties and that properly accumulated and delivered both drugs in damaged joints. As a consequence, these structures had significant anti-inflammatory effects by reducing the concentrations of joint nitric oxide (NO) and the expression of M1 macrophage markers, while enhancing that of M2 macrophage markers, following rapid uptake by the macrophages. This may favor their retention at inflamed locations by the extravasation through leaky vasculature and subsequent inflammatory cell-mediated sequestration effect (ELVISE).

Tofacitinib (TFC) is another candidate for RA therapy as a novel, oral non-traditional Janus kinase (JAK) inhibitor with similar efficacy and safety to that of other DMARDs [111]. However, its clinical application has been hindered thus far by its low plasma half-life. Bashir et al. [112] designed innovative PLGA-based NPs to promote the sustained release and target-specific delivery of TFC. PLGA is one of the most commonly employed biocompatible and biodegradable polymers that is applied in various drug delivery systems. Hydrolization of PLGA produces two main monomers in water, e.g., poly-lactic acid (PLA) and poly-glycolic acid (PGA). The authors encapsulated TFC in PLGA NPs by nanoprecipitation as a new nanodelivery structure to target the inflamed synovium and demonstrated that such TFC-PLGA-NPs supported an adapted TFC pharmacokinetic profile [112].

**Figure 3:**
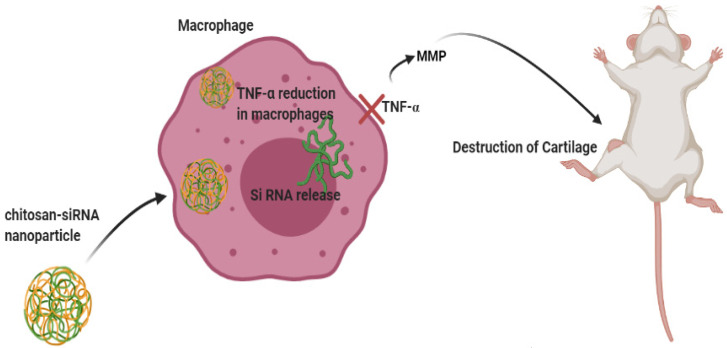
CS-siRNA NP delivery in macrophages to suppress TNF-α expression. Macrophage elimination by CS-siRNA NPs was established to inhibit a local production of IL-1β, IL-6, TNF-α, and matrix metalloproteinases (MMPs) and thus reduce the pathogenesis of inflammatory arthritis.

A study by Howard et al. [113] investigated the impact of an CS-siRNA NP so suppress inflammatory TNF-α expression in macrophages in CIA mice. The histological examination of the joints indicated slight cartilage damage and inflammatory cell penetration in anti-TNF-α-treated mice, demonstrating the benefits of such a TNF-α knockdown approach via CS-siRNA NP on local and systemic inflammation (Figure 3).

Lee et al. [99] adopted a similar approach using nanostructure polymerized siRNA (poly-siRNA) targeting TNF-α with thiolated glycol CS (TGC) polymers to control the progression of RA in mice. TNF-α expression was downregulated in vitro via the application of these NPs and at arthritic sites in RA mice joints. Ha et al. [114] investigated the potential of photothermally organized drug delivery using multifunctional NPs (MNPs) with a NIR irradiated location, with improved therapeutic efficacy for patients suffering from RA and decreased side effects. In this approach, the layer of Au film was applied to MTX-encapsulated PEG-PLGA NPs, allowing for correct encapsulation of MTX in the MNPs. The synergistic interactions of MTX-encapsulated MNPs accompanied by NIR rays were tested in fibroblast-like RA synoviocytes (FLSs) and CIA mice [114]. The advantage of using NIR resonance of the Au shell is to accelerate the local release of MTX from the NPs. NIR images of MTX-loaded MNPs showed suitable transfer of the MNPs to the inflamed joints. Furthermore, repeated administration of MNPs encapsulating MTX at a 1/1400 solution had stronger anti-RA effects in CIA mice than the MTX solution itself. Furthermore, combining MTX-encapsulated MNPs with NIR radiation was more efficient than chemotherapy alone. Overall, MTX-loaded MNP therapy with NIR irradiation represents a suitable treatment for RA based on a single, low dose of MTX [114].

### 3.3. Niosomes

**Figure 4:**
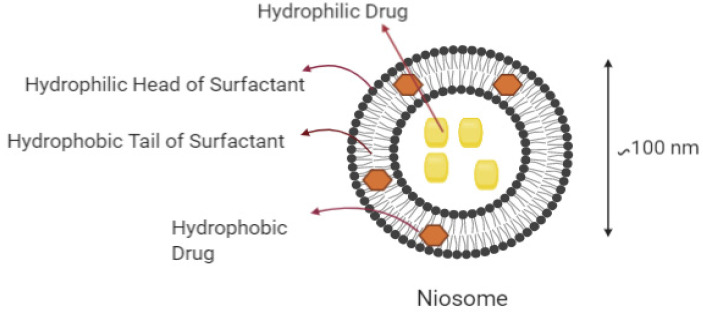
Structure of niosomes for the transfer of various drugs to target locations.

Niosomes are vesicles with lamellar morphology that are microscopic in size and are mostly composed of nonionic surfactants and cholesterol (Figure 4). They display significant advantages as drug delivery systems due to their ability to support a controlled release of drugs at a targeted site, to their nonimmunogenic properties and biocompatibility, and to their delayed clearance from the environment [115].

Niosomes can be prepared when nonionic surfactant vesicles self-associate together. Several factors affecting the formation of niosomes include the type of non-ionic surfactant used, the hydration temperature, and the preparation method [116]. Primary surfactant vesicles are generally made of ionic surfactants. Work showed that the highest toxicity is associated with cationic surfactants followed by anionic surfactants, with nonionic surfactants displaying the lowest toxicity. For these reasons, niosomes made from nonionic surfactants are ideal choices for transdermal delivery. Hydrophilic–lipophilic balance (HLB) values play a significant role in the preparation of niosomes and a range of HLB 4–8 is usually used. Common surfactants used to make niosomes include Span, Brij, and Tween [117,118].

In recent study, Rajaram et al. [119] designed a promising system to deliver piroxicam (PC) encapsulated in a vesicular carrier of nonionic surfactant as transdermal patches, a suitable drug delivery system to increase the solubility of drugs with poor solubility and to increase the retention time of the drug at the site of absorption. This approach may be a useful structure for treatment of RA. Another report from Paradkar et al. [100] showed the possibility to prepare a niosomal thiocolchicoside topical gel using the thin film hydration procedure, consisting of a molar ratio of span 60:cholesterol. This niosomal gel increased the topical retention time and controlled the pain caused by RA and its side effects at reduced dosing frequency. Mujib et al. [120] designed a study to treat RA with the aim of increasing the duration of drug action at lesser side effects. Topical gel formulations containing ibuprofen loaded in niosomes were prepared by changing the ratios of various nonionic surfactants (span20, span60, span80, and cholesterol) by the thin film hydration and ether injection methods. The results indicated that the niosomal nanocarrier of ibuprofen gel containing carbopol acted as the basis of an appropriate topical drug delivery system for lagging the duration of the drug.

### 3.4. Metal NPs

The unique biological, physical, mechanical, chemical, and thermal features of metal NPs made them increasingly used systems in the medical and pharmaceutical fields [52]. Metal NPs are very small mineral particles with at least one dimension in a size range below 100 nm [121].

#### 3.4.1. Silver NPs

Silver NPs (Ag NPs) have been reported for their anti-inflammatory activities by limiting the production of proinflammatory cytokines such as TNF-α and IL-6 and have been beneficial in RA patients [122]. Ag NPs can also reduce the amount of vascular endothelial growth factor (VEGF), a factor produced by epithelial cells that increases antigen sensitivity, exhibits a major function in physiological abnormalities, causes plasma proteins to leak into the extracellular space, leads to a thickening of the airway wall, and increases the T helper type-2 (TH2) cell-mediated inflammation (IL-9, IL-4, IL-5, and IL-13) [123]. Ag NPs inhibit the Src kinase pathway and block Y419 phosphorylation in a dose-dependent manner, reducing the vascular endothelial permeability induced by VEGF and IL-1β. Ag NPs also can block VEGF and IL-1β-induced solute flux and decrease VEGF-induced cell production [123]. Ag NPs can also decrease the expression of the hypoxia-induced factor 1 alpha (HIF-1α) that regulates the expression of proinflammatory genes and kill bacteria [123]. Finally, Ag NPs can restrict the secretion of proinflammatory mediators such as TNF-α, IL-12, and COX-2 at higher concentrations [25].

M1 macrophages play key roles during the pathogenesis of RA. Infiltration of these inflammatory cells leads to the secretion of different inflammatory cytokines. To improve synovial inflammation, M1 macrophages may progress to an M2 anti-inflammatory macrophage phenotype [124]. Yang et al. [124] used folic acid modified with silver nanoparticles (FA-AgNPs) which could be effectively transported into M1 macrophages to stimulate M1 macrophages reduction and then M2 macrophages polarization in order to successful RA therapy. In this model, M1 macrophages were targeted through the folate receptor overexpressed at their cell surface. After entering the cell, these NPs dissolve in response to intracellular glutathione and release silver ions. In fact, this process is a key factor in starting a series of anti-inflammatory actions like the removal of reactive oxygen species (ROS) to accelerate the polarization of M2 macrophages. These NPs accumulate passively in inflamed joints, having strong anti-inflammatory activities and therapeutic effects in mice model of RA with high safety.

#### 3.4.2. Gold NPs

An et al. [125] prepared Au NPs from aqueous leaf extracts of *M. alliacea* with a face-centered cubic form as reported by X-ray diffraction (XRD) and a diameter of 20–30 nm as noted by transmission electron microscopy (TEM). The effects of gold were determined using tail-flip examination, a sensitive drug model that operates in the central nervous system (CNS). A maximum heat threshold of almost 3.5 times that of the treated vehicle was observed 2 h after treatment. Such Au NPs were capable of reversing Complete Freund’s Adjuvant (CFA)-induced thermal hyperalgesia that is detected in physiological conditions. In another study, investigators showed that the reaction between synoviocytes and macrophages was effectively involved in causing inflammatory responses in the synovium. The results indicated that triamcinolone-gold NPs (Triam-Au NPs) increased the anti-inflammatory reactions of FLSs and macrophages through the repolarization of macrophages from an M1 to an M2 phenotype and reduced proinflammatory responses. Triam alone reduced the proinflammatory reactions of FLSs and macrophages. Different experiments in various test conditions in vitro/ex vivo (human samples) and in vivo (mice) revealed that Triam-Au NPs effectively repolarized macrophage activity in damaged synovium and improved the appearance of cartilage tissue while Triam alone did not prompt FLS anti-inflammatory actions nor macrophage repolarization [126].

#### 3.4.3. Iron NPs

Some researchers assessed the intra-articular adsorption rate of super-paramagnetic iron oxide nanoparticles (SPIONs) covered with poly-vinyl-alcohol (PVA-SPIONS) by the synovial membrane in an animal model in vivo. The NPs remained in the synovium for almost a week, demonstrating that such systems may provide an effective platform for intra-articular drug delivery especially for the treatment of acute or chronic joint diseases [99].

SPIONs are appropriate nanocarriers with an extensive ability for drug delivery and can be conjugated with several therapeutic agents for controlled release to a target site by an external magnetic field. Carneiro et al. [127] prepared a system of magnetic targeting with using gold-covered superparamagnetic iron oxide nanoparticles (Au-SPIONs) to target the joints of CIA rats. Such a system improved RA signs without undesirable side (toxic) effects. Similar to this approach, a study published in 2019 showed that magnetic targeting of Au-SPIONs remained longer in joints than colloidal Au-NPs and had greater antioxidant effects than MTX. In this method, Au-SPIONs were intra-peritoneally administered in collagen-induced arthritis (CIA) rats three times daily. After that, a neodymium magnet was coupled to the right ankle joint for 1 h targeting. The results showed that the Au-SPIONs significantly reduced tissue edema, leukocyte infiltration, and TNF-α and IL-1β expression in the synovial fluid of CIA [39]. In another study, MTX and SPIONs were co-encapsulated in to the SLNs in order to be utilized as therapeutics and imaging factors, respectively. Transmission electron microscopy (TEM) images showed that the SPIONs were loaded in to the SLN matrix, and MTX encapsulation efficiency amounts were more than 98%. In vitro investigations demonstrated that the formulations exhibited minor toxicity at concentrations lower than 500 μg/mL, by using THP-1 cells. Moreover, these SLNs have been functionalized with anti-CD64 which is a kind of antibody that specially attached to a surface of cell receptor over expressing in RA infected macrophages. The results indicated that the NP structures offered suitable and promising outcomes for future theranostics of RA. Moreover, the proposed nanoformulations are very hopeful for the future of RA therapy and diagnostic, because of a nontoxic and targeted delivery of drugs and simultaneously permitting in vivo imaging [128].

### 3.5. Quantum Dots

Quantum dots (QDs) are inorganic nanomaterials or semiconductor nanocrystals with a size of 1–10 nm, consisting of a central semiconductor core with a shell consisting of inorganic salts (CdS, ZnS).

QDs have been employed in several nanotechnological procedures including for drug delivery, bioimaging of abnormal cells, and RA diagnosis and treatment. Kalangi et al. [129] tested mercaptopropionic acid (MPA) nanoconjugates surrounded with Cd-Te QDs and celecoxib (a strong COX-2 inhibitor) for bioimaging in Carrageenan-induced mice with paw inflammation. The authors demonstrated the ability of QD-celecoxib conjugates as substitutes of radioisotopes to reveal the localization of the drug in sites of inflammation in this animal model and may be of further help to evidence off-targets.

### 3.6. Solid Lipid NPs

SLNs are novel and interesting pharmaceutical drug delivery platforms with a diameter of 120–200 nm that are extensively used for managed drug delivery, combining the advantages of polymeric NPs and oil-in-water emulsions [122].

Among various drugs, anti-inflammatory CUR has been reported for its benefits in a wide diversity of inflammatory diseases, but its use may be limited by its weak absorption, fast metabolism, and rapid systemic elimination. To overcome such issues, Arora et al. [130] evaluated the potential of a CUR-SLN system to manage joint hyperalgesia and stiffness with enhanced leukocyte count, TNF-α, CRP, and oxidative stress in CFA-induced inflammation in rats relative to free CUR treatment. They observed a significant, dose-dependent reduction of RA signs in the animals upon CUR-SLN administration compared with free CUR application via decreased oxido-inflammatory and immunomodulatory signals. In another study, Bhalekar et al. [131] encapsulated piperine (Pi) in SLNs (Pi-SLNs) using the melt emulsification technique to treat RA. Pi is an alkaloid substance originates from the fruits and also roots of Piper nigrum types of Piperacea group. The authors reported an average particle size of 128.80 nm with a zeta potential of −23.34 mV and an encapsulation efficacy of 78.71%. The prepared SLN was administered to CFA-induced inflammatory mice orally and topically. In the study, the Franz diffusion cell indicates that Pi from the SLN gel formulation assembles in the skin. The outcomes of the pharmacodynamic investigation show that both topical and oral Pi have a suitable response compared to for example oral chloroquine suspension. Alarifi et al. [132] designed formulations of ciprofloxacin (CIP) into SLNs as a means to improve its biopharmaceutical and biochemical properties and its anti-inflammatory properties. The results of the evaluation showed that encapsulation of CIP in SLNs increased its antimicrobial and anti-inflammatory activities in vitro which could significantly improve a treatment by reducing dose-dependent side effects and increasing the range of protective activities.

### 3.7. Polymeric Micelles

Biocompatible and biodegradable polymers are widely used in pharmaceutical processes as useful components for pharmaceutical formulations and also utilized as nanocarriers in drug delivery systems [133]. Currently, micelles are based on the use of amphiphilic block copolymers in aqueous solution. They can be employed as nanocarriers for less soluble drugs that may be covalently attached to polymer chains or non-covalently placed in micelles [133]. Generally, they are suitable nanovehicles to improve several biochemical and biopharmaceutical properties such as solubility, bioavailability, and targeting of these hydrophobic drugs. They are adapted for use in combination with other therapeutics agents for RA remedy. Twenty years ago, an outstanding novel micelle-based system has been developed for the treatment of RA [134].

Glucocorticoids (GLCs) are among the most critical agents used in RA, but they display serious side effects when applied at high doses. In this regard, Wang et al. [135] applied GLCs in a rat model of arthritis using a micellar nanosystem, allowing for a targeted, low-dose drug delivery in the damaged joints capable of safely ameliorating their therapeutic efficacy. Micelles loaded with Dex self-assembled from the amphipathic PEG-block-poly(caprolactone) (PEG-PCL) polymer through film dispersion were intravenously injected in adjuvant-induced arthritis rats using only a dose of 0.8 mg/kg. The micelles remained in the circulation for a significant period of time and then accumulated in the inflamed joints. Micelle-delivered Dex decreased joint pain and swelling as well as proinflammatory cytokine expression in both the joint tissues and the blood. PEG-PCL micelles led to not only reduced adverse effects on body weight, but also decreased the lymphocyte counts and the blood glucose levels. These findings show that loading Dex in PEG-PCL micelles may allow for a suitable and effective low-dose GC therapy targeting inflammatory diseases. Abdollahi et al. [136] prepared novel dextran stearate polymeric micelles by the dialysis method as a means to transport indomethacin, a NSAID that can successfully decrease the RA’s pain and inflammation, in order to decrease its otherwise serious side effects. Such micelles delivered a more impact and useful drug that reduced the inflammation in a rat model of arthritis compared with the treatment with indomethacin alone.

## 4. Conclusions, Challenges, and Future Opportunities

RA is an inflammatory and chronic disorder related to abnormal function of immune system. In RA, the immune system mainly attacks the joints, causing their inflammation like in the knee, wrist, and hands. This tissue damage can cause long-term pain and improper deformation of the body. In several patients, RA can damage a wide part of our body, including the skin, eyes, hearts, lungs, and blood vessels. The primary goals of treating RA are to control the inflammation and pain and to slow or stop the progression of the disease. Early treatment approaches include MTX and sulphasalazine which suppress the immune system. Although these drugs are appropriate, suppressing the immune system increases the risk of infection. It is also critical to keep in mind the potential side effects of the drugs, like nausea, abdominal pain, and severe damage to the liver and the lungs. As the effects of these drugs usually last 6–12 weeks, rheumatologists may also administrate them with NSAIDs to treat pain and inflammation. Despite these issues, DMARDs are still used as first-line therapies. In this article, our goal was to describe several types of nanomaterials for applications in the treatment of RA. The results of this review show that all nanocarriers have unique properties to encapsulate anti-RA drugs and reduce drug side effects. The evaluations also showed that these systems can increase the bioavailability of hydrophobic drugs and enhance their effectiveness for RA. Briefly, the present review discusses several useful and successful nanocarriers in order to exactly delivery several therapeutic agents to target sites in RA therapy. Table 1 shows various nanostructures as a novel platform in improving function of anti-RA drugs.

The expanded use of nanomaterials in a wide variety of biomedical applications, on the other hand, also raises questions about their toxicity. The morphological and physicochemical properties of nanomaterials play an important role in determining their toxicity in various body organs such as the liver, kidney, skin, brain, heart, etc. While nanomaterials have significant toxicological effects in various experimental models, their toxicity can be minimized or eliminated by engineering their surface with various types of natural or synthetic polymers and other compounds. While some work explored the toxicity of nanomaterials for biomedical application, there is thus far no in-depth information available on these aspects.

## Figures and Tables

**Table 1:** Various nanostructures as a novel platform in improving function of anti-RA drugs.

Nanocarriers	Drugs	Effects	References
Liposomes	MTX, Dex, SIN	Improved controlled release, reduced RA signs, decreased side effects	[104]
Polymeric NPs	Ket, Dex, TFC, MTX	Decreased inflammation	[110]
Niosomes	PC, thiocolchicoside, ibuprofen	Increased drug retention	[119]
Silver NPs	FA-AgNPs	Enhanced anti-inflammatory activities	[124]
Gold NPs	Triam	Enhanced anti-inflammatory activities	[126]
Iron NPs	MTX	Suppression of joint edema and inflammation	[128]
Quantum dots	celecoxib	Localized activity in sites of inflammation	[129]
Solid lipid NPs	CUR, Pi, CIP	Enhanced anti-inflammatory activities	[130]
Polymeric micelles	Dex, indomethacin	Decreased side effects	[135]

Abbreviations: NPs, nanoparticles; MTX, methotrexate; Dex, dexamethasone; SIN, sinomenine hydrochloride; Ket, Ketoprofen; TFC, tofacitinib; PC, piroxicam; FA-AgNPs, folic acid modified with silver nanoparticles; Triam, triamcinolone; CUR, curcumin; Pi, piperine; CIP, ciprofloxacin; RA, rheumatoid arthritis; COX-2, cyclooxygenase 2.

## Data Availability

Not applicable.
