# Correction: Hosseinikha et al. Nanomaterials for the Diagnosis and Treatment of Inflammatory Arthritis. *Int. J. Mol. Sci.* 2021, *22*, 3092

**DOI:** 10.3390/ijms26094141

**Published:** 2025-04-27

**Authors:** Seyedeh Maryam Hosseinikhah, Mahmood Barani, Abbas Rahdar, Henning Madry, Rabia Arshad, Vahideh Mohammadzadeh, Magali Cucchiarini

**Affiliations:** 1 Nanotechnology Research Center, Pharmaceutical Technology Institute, Mashhad University of Medical Sciences, Mashhad 91886-17871, Iran; mhosseinikhah@yahoo.com; 2 Department of Chemistry, Shahid Bahonar University of Kerman, Kerman 761691411, Iran; 3 Department of Physics, Faculty of Science, University of Zabol, Zabol 538-9861, Iran; 4 Center of Experimental Orthopaedics, Saarland University Medical Center, D-66421 Homburg/Saar, Germany; henning.madry@uks.eu; 5 Department of Pharmacy, Quaid-i-Azam University, Islamabad 45320, Pakistan; rabia.arshad@bs.qau.edu.pk; 6 Department of Pharmaceutical Nanotechnology, School of Pharmacy, Mashhad University of Medical Science, Mashhad 91886-17871, Iran; mohammadzadehv971@mums.ac.ir

In the original publication [1], the author Abbas Rahdar’s email is replaced with a.rahdarnanophysics@gmail.com.

Following the publication, concerns relating to some citations were brought to the attention of the authors and the Editorial Office.

Adhering to our complaints procedure, an investigation was conducted by the authors, the Editorial Office, and Editorial Board, which confirmed that the references [49–72] include a certain number of self-citations [1], which is against our citation policies (https://www.mdpi.com/ethics#_bookmark20). As a result, the authors, the Editorial Office, and Editorial Board have decided to remove the references [49–72] in Section 1 as per MDPI’s cor.96rection policy (https://www.mdpi.com/ethics#_bookmark30). References [73–160] in the original publication were updated to [49–136] accordingly.

The authors state that the scientific conclusions are unaffected. This correction was approved by the Academic Editor. The original publication has also been updated.
